# Time to recovery from moderate acute malnutrition and its predictors among children aged 6–59 months in Fedis Woreda, East Hararghe Zone, Eastern Ethiopia

**DOI:** 10.3389/fnut.2024.1369419

**Published:** 2024-08-07

**Authors:** Berhanu Melaku, Berhe Gebremichael, Belay Negash, Monas Kitessa, Obsan Kassa, Jerman Dereje, Reta Kefelegn, Dawit Firdisa

**Affiliations:** ^1^School of Public Health, College of Health and Medical Sciences, Haramaya University, Harar, Ethiopia; ^2^School of pharmacy, College of Health and Medical Sciences, Haramaya University, Harar, Ethiopia; ^3^Department of psychiatry, School of Nursing and Midwifery, College of Health and Medical Sciences, Haramaya University, Harar, Ethiopia

**Keywords:** time to recovery, moderate acute malnutrition, targeted supplementary feeding program, East Hararghe, Eastern Ethiopia

## Abstract

**Background:**

Acute malnutrition is a major global public health problem, particularly in low-and middle-income countries. A targeted supplementary feeding program is an approach recommended to address moderate acute malnutrition in food-insecure settings. Preventing and treating moderate acute malnutrition requires identifying factors shown to affect the treatment outcome and duration of stay on treatment. This study aimed to determine the time to recovery from moderate acute malnutrition and its predictors among children aged 6–59 months in Fedis Woreda East Hararghe Zone, Eastern Ethiopia, from January 1 to December 31, 2022.

**Methods:**

A facility-based retrospective cohort study was conducted on 567 children with moderate acute malnutrition in Fedis Woreda, East Hararghe Zone, eastern Ethiopia. A multi-stage sampling technique was employed, and data was collected using a structured checklist. Data were extracted from randomly selected records after obtaining ethical clearance. Data were cleaned, coded, entered into EpiData 4.6, and analyzed using STATA/SE version 14. Descriptive statistics and analytic analysis schemes, including bivariable and multivariable Cox proportional hazards models, were conducted, and finally, statistical significance was considered at *p* < 0.05.

**Results:**

The overall median time to recovery was 16 weeks. The major predicting factors for time to recovery among children aged 6–59 months were admission with a mid-upper arm circumference of 12.1–12.4 centimeters (AHR = 1.02, 95% CI: 1.01–1.19), access to transportation to facilities (AHR = 0.62, 95% CI: 0.36–0.81), children using specialized nutritious foods (RUSF; AHR = 1.96, 95% CI: 1.36–3.11), and children who had diarrhea (AHR = 0.4, 95% CI: 0.31–0.71).

**Conclusion:**

The study found a median recovery time of 16 weeks for children with targeted supplementary feeding. Significant predictors included admission with a MUAC of 12.1–12.4 centimeters, transportation access, RUSF use, and the presence of diarrhea. These findings highlighted the importance of these factors in determining and improving recovery from moderate-acute malnutrition.

## Introduction

Malnutrition refers to deficiencies or excesses in nutrient intake, an imbalance of essential nutrients, or impaired nutrient utilization (1). Acute malnutrition is one of the three main types of under-nutrition, affecting potentially all categories of the population but especially vulnerable groups such as children under five, pregnant and lactating women, and people living with a disease or chronic illness (2). Acute malnutrition results from a sudden reduction in food intake or diet quality, and it is often combined with pathological causes. It is classified as moderate acute malnutrition (MAM)/ wasted or severe acute malnutrition (SAM)/ severely wasted (3). Severe acute malnutrition is defined as a very low weight for height (WFH) below -3z scores of the median WHO growth standards, and/ or MUAC <11.5 cm, and/ or presence of nutritional edema (4). Moderate acute malnutrition (MAM), defined as having a weight-for-height Z-score (WHZ) −3 and − 2, without edema and/or mid-upper arm circumference (MUAC) 11.5 to <12.5 cm (5). It is associated with increased mortality and morbidity among children under 5 years of age (6, 7).

Children with MAM face a greater risk of morbidity from infectious diseases, weakened immune systems, and impaired physical and cognitive development (7–9). Moreover, beyond deterioration to severe acute malnutrition, a recent study suggested that repeated episodes of moderate acute malnutrition in children can eventually lead to stunting over time with irreversible inter-generational growth failure (10).

Targeted Supplementary Feeding is implemented in food insecure situations, including in emergencies, in order to treat MAM and prevent children with MAM from becoming severely malnourished (falling into SAM). It is usually indicated when MAM and SAM prevalence rates are between 10 and 14% or 5–9% with aggravating circumstances (11). In Ethiopia, 7% of children 6–59 months of age are wasted, with 70% of these having MAM, and about 35 to 57% of the deaths in under-five children are attributable to malnutrition (12). Malnutrition among children is a critical problem because its effects are long-lasting, go beyond childhood, and affect academic performance, physical development, and mental development throughout their lives (13).

Globally, an estimated 47 million children younger than 5 years had acute malnutrition, of which 70% were attributed to moderate acute malnutrition (14). Africa and Asia bear the greatest share of moderate-acute malnutrition (7). Acute malnutrition prevalence varies across regions and countries, with a high burden remaining in low-and middle-income countries, with an estimated figure of 7.7, 2.0, and 0.7% in South Asia, Sub-Saharan Africa, and Latin America, respectively (15). Ethiopia has made substantial progress in reducing the prevalence of malnutrition in the past two decades (16, 17). However, childhood under-nutrition, particularly moderate acute malnutrition, remains a major challenge (18). Moreover, the progress made has been uneven across the administrative regions of Ethiopia, as the prevalence of moderate acute malnutrition ranges from the highest of 21% in the Somali region to the lowest of 2% in Addis Ababa (19).

Previous studies conducted in various regions of Ethiopia have been focused on the management of severe acute malnutrition (20–23). However, the management of moderate acute malnutrition should also be a public health and development priority (24). Preventing and treating MAM requires understanding the factors shown to affect the management outcomes and duration of stay on treatment (25). Though the recovery time of MAM is within a wide statistical range, no study reported MAM treatment outcomes, particularly in the eastern part of Ethiopia. This study seeks to shed light on the time to recovery from MAM and its predictors among children aged 6–59 months in Fedis Woreda, East Hararghe Zone, eastern Ethiopia.

## Materials and methods

### Study design, and setting

This study employed an institution-based retrospective cohort study and was conducted from January 1 to December 30, 2022, in Fedis woreda, East Hararghe Zone, Oromia region, located 541 km to the east of Addis Ababa and 24 km to the south of Harar Town.

Fedis Woreda is situated in the East Hararghe Zone of eastern Ethiopia. The district has an estimated population of 168,770. To address food insecurity, Fedis Woreda has implemented targeted and blanket food supply programs. The health infrastructure consists of five government health centers and 21 health posts, all offering services for children with moderate acute malnutrition (MAM) and pregnant and lactating women (PLW). Data for this study was extracted from February 1, 2023, to March 30, 2023.

### Population and sampling

The study population consisted of children aged 6 to 59 months who were diagnosed with moderate malnutrition and enrolled in the targeted supplementary feeding (TSF) program at selected health posts in Fedis Woreda from January 1 to December 30, 2022. Records of all eligible 6-59-month-old children who were identified from the registration book and identified to have moderate acute malnutrition were included in the study, whereas records with incomplete information like missed age, sex, and MUAC were excluded from four health posts.

The sample size was determined using both a single and double population proportion formula. The maximum sample was achieved by using a double proportion formula by considering the assumptions: 95% confidence level, 80% power, one-to-one ratio, proportion [p1 = 43.8%, p2 = 56.2% (26), and 10% of non-respondents]. The final sample size was 567.

In the study area, 8 out of the 21 health posts in the woreda were randomly selected using a lottery method to ensure representativeness. The assumption was made that the populations around these health posts were relatively homogeneous. The management protocol for moderate acute malnutrition (MAM) was standardized across all health post-levels in the woreda. A sampling frame was created for children undergoing MAM management in the eight selected health posts. Probability proportional to size sampling was used to allocate samples to each health post based on their population size.

Finally, children were selected for the study through simple random sampling within each health post, using their unique identification numbers, to ensure a representative sample from the selected institutions.

### Data collection methods and quality control

Data for the study was extracted from the registration book using a well-designed data extraction form. Data were collected by eight trained health extension workers and supervised by two BSc nurses. The records of eligible children were retrieved from the registration book, and their individual cards were reviewed. Relevant information, including admission medical history, physical examination findings, follow-up anthropometry measurements, clinical features, routine medications, and outcome status, was recorded.

A pre-test of the data collection tool was conducted in the Fedis district. Completed questionnaires were checked for completeness and consistency. Data cleaning was done daily, and then feedback was given to data collectors. The principal investigator supervised secondary data collection from the beneficiary registration book to ensure accuracy. The collected data was carefully reviewed to ensure completeness and consistency. Each questionnaire received a unique code for identification.

### Data processing and statistical analysis

Data entry was performed using EpiData 4.6 and checked for errors and missing values. Subsequently, the data was exported to Stata version 14.0 for further analysis. Descriptive statistics such as frequencies, percentages, and summary measures were done to describe the variables of interest.

In the study, the Life table method was utilized to estimate the probabilities of recovery. Both bivariate and multivariable Cox proportional-hazard regression models were done to identify predictors of the time to recovery. Independent variables with a *p*-value of less than 0.25 in the bivariate analysis were included in the final multivariate model. The results were reported as adjusted hazard ratios (AHR) with a 95% confidence interval, indicating the presence, strength, and direction of the association between the predictors and the time to recovery.

## Results

### Socio demographic characteristics of participants

The study included a sample of 567 children aged 6 to 59 months with moderate acute malnutrition from eight health posts in Fedis Woreda, Eastern Hararghe zone. The average age of the children in the program was 16.48 months, with a standard deviation of 18 months. Among the participants, 285 (50.3%) were male, and 432 (76.2%) resided in rural areas. The primary caregivers for the children were predominantly mothers, accounting for 95% of the cases (Table 1).

**Table 1:** Socio-demographic characteristics of children 6–59 months children in Fedis Woreda, east Hararghe, Ethiopia, 2022.

Variables	Category	Frequency	Percentage (%)
Age in months	6–23	295	52.1
	24–59	272	47.9
Sex of the child	Male	285	50.3
	Female	282	49.7
Place of residence	Rural	432	76.2
	Urban	135	23.8
Primary care giver	Mother	541	95
	Father and Other (Specify)	26	5

### Family and health care related characteristics of study populations

Among the participants, 87.5% of them recovered from the program, while 12.5% did not. Also, 60% of participants were admitted with a MUAC measurement of 11.5–12.0 cm and 40% with 12.1–12.5 cm. Additionally, 93.6% used ready-to-use supplementary feeding, and 6.4% used Super Cereal Plus. 60% received vitamin A supplementation and 40% did not; 87.8% were new admissions and 12.2% were re-admissions; 77.1% traveled over 60 min to the distribution center; and 22.9% traveled less than 60 min. Further, 90.7% of caregivers received health and nutritional education, while 9.3% did not. Among the children, 9% had diarrhea, with 90.7% experiencing watery diarrhea and 8% experiencing bloody diarrhea (Table 2).

**Table 2:** Family and healthcare related characteristics of study population of time to recovery from moderate acute malnutrition among 6–59 months children in Fedis Woreda, east Hararghe, Ethiopia, 2022.

Variables	Category	Frequency	Percentage
MUAC at Admission(cm)	11.5–12.0	340	60
	12.1–12.4	227	40
Vitamin A supplementation	Yes	478	84.3
	No	89	15.7
Deworming	Yes	166	29
	No	401	71
Admission status	New admission	498	87.8
	Re admission	69	12.2
Distance from facility in minutes	<60	437	77.1
	≥60	130	22.9
Transportation access to nearest health post and food distribution center	Yes	333	58.7
	No	234	41.3
Child received measles vaccination	Yes	396	70
	No	171	30
Caregiver get education during distribution period	Yes	514	90.7
	No	53	9.3
Cooking demonstration conducted during distribution period	Yes	486	85.7
	No	81	14.3
Specialized nutritious foods given to the child	CSB++	36	93.6
	RUSF	531	6.4
Number of sachets of CSB++ is given for a child per month	Less than three	0	0
	Three	0	0
	Four	36	100
	More than four	0	0
Number of plumpy sup given to a child per day	One	553	97.5
	Two	14	2.5
Fever	Present	82	14.5
	Absent	485	85.5
Hypothermia	Present	3	0.5
	Absent	464	99.5
Pneumonia	Present	46	9.1
	Absent	521	91.9
Vomiting	Present	38	6.8
	Absent	529	93.2
Diarrhea	Present	51	9
	Absent	516	91
Type of diarrhea	Watery diarrhea	47	92
	Dysentery	40	8
Duration of diarrhea (days)	2	20	39.2
	3	18	35.3
	4	7	13.7
	5	6	11.8
Anemia	Yes	8	1.4
	No	559	98.6

### Time to recovery from moderate acute malnutrition among 6–59 months children

In a study involving 567 children with moderate acute malnutrition (MAM) enrolled in targeted supplementary feeding, 87.5% (496) of the children successfully recovered within a median time of 16 weeks (interquartile range: 5.4). However, 12.5% (71) did not recover. The study found that male children had a shorter median recovery time (15 weeks, interquartile range: 6) compared to female children (17 weeks, interquartile range: 7). Furthermore, the analysis revealed that children aged 24–59 months were 1.30 times more likely to recover from MAM within 16 weeks (adjusted hazard ratio: 1.30, 95% CI: 1.11–1.56) compared to those aged 6–23 months. Additionally, female children with MAM had an 8% lower likelihood of recovery within 16 weeks compared to male MAM children (Table 3).

**Table 3:** Life table of time to recovery of MAM children at different week among 6–59 months children in Fedis Woreda, east Hararghe, Ethiopia, 2022.

Interval start time	Number Entering Interval	Number Withdrawing during Interval	Number Exposed to Risk	Number of Terminal Events	Proportion Terminating	Proportion Surviving	Cumulative Proportion Surviving at End of Interval	Hazard Rate
0	567	0	567.000	0	0.00	1.00	1.00	0.00
4	567	0	567.000	0	0.00	1.00	1.00	0.00
8	567	0	567.000	102	0.18	0.82	0.82	0.06
12	447	4	445.000	154	0.35	0.65	0.53	0.18
16	245	1	244.000	53	0.63	0.37	0.2	0.30
20	91	0	91.000	84	0.92	0.08	0.02	0.46
24	13	0	13.000	13	1.00	0.00	0.00	0.00

### Predictors of time to recovery

This study examined several factors associated with the time to recovery of moderate acute malnutrition (MAM) treatment. The bivariate analysis revealed significant associations between the primary child care responsibility, admission MUAC, Vitamin A supplementation, admission status, transport access to the nearest TSFP distribution center, measles vaccination, types of treatment food, and co-morbidities such as fever and diarrhea, with the dependent variable (time to recovery) at a *p*-value of less than 0.25 (Table 4). Furthermore, the multivariate Cox regression analysis identified admission MUAC, transport access to the nearest health facility, types of specialized food, and diarrhea as significant predictors of the time to recovery in MAM treatment, with a p-value of less than 0.05 (Table 5).

**Table 4:** Bivariate cox-regression of predictors of time to recovery from moderate acute malnutrition among 6–59 months children in Fedis Woreda, east Hararghe, Ethiopia, 2022.

Variables	Category	No.	CHR	*p*-value
Age of the children	6–24	295	1	1
	25–59	272	1.30(1.11–1.56)	0.38
Sex of the children	Male	285	1	1
	Female	282	0.92(0.81–1.2)	0.47
Place of residence	Urban	135	1	1
	Rural	432	0.71(0.46–1.81)	0.59
Person with primary Child responsibility	Mother	541	1	1
	Father	26	1.64 (0.92–3.3)	0.19
MUAC at Admission	11.5–12.0	340	1	1
	12.1–12.4	227	1.34(0.88–1.54)	0.21
Vitamin A	Yes	478	1	1
	No	89	0.81(0.64–1.02)	0.23
Deworming	Yes	166	1	1
	No	401	1.14(0.88–1.31)	0.78
Amoxicillin	Yes	162	1	1
	No	405	1.49(0 0.96, 1.68)	0.62
Admission status	Yes	498	1	1
	No	69	1.52(0.81–3.01)	0.18
Distance from facility in minutes	<60	437	1	1
	> = 60	130	0.89(0.71–1.25)	0.51
Transportation access to nearest health post and food distribution center	Yes	333	1	1
	No	234	0.73(0.52–0.89)	0.014
Child received measles vaccination	Yes	396	1	1
	No	171	0.65(0.51–1.93)	0.15
Caregiver get education during distribution period	Yes	514	1	1
	No	53	1.12(0.55–2.20)	0.87
Cooking demonstration conducted during distribution period	Yes	486	1	1
	No	81	0.91(0.83–1.21)	0.66
Specialized nutritious foods given to the child	CSB	36	1	1
	RUSF	531	1.82(1.31–2.32)	0.01
Number of sachets of CSB++ is given for a child per month?	Four	36	1	1
	Less or more than four	0	1.26(0.93–1.84)	0.75
Number plumpy sup given to a child per day	One	553	1	1
	Two	14	0.75(0.64–1.08)	0.42
Fever	Yes	82	1	1
	No	485	2.1(0.92–7.45)	0.19
Hypothermia	Yes	3	1	1
	No	464	1.9 (0.35–2.5)	0.64
Pneumonia	Yes	46	1	1
	No	521	2.46 (0.7–9.3)	0.56
Vomiting	Yes	38	1	
	No	529	2.01(0.45–6.7)	0.91
Diarrhea	Yes	51	1	1
	No	516	1.33(0.93–2.01)	0.12
Anemia	Yes	8	1	1
	No	559	2.11(0.87–8.55)	0.72

**Table 5:** Multivariable cox-regression analysis for predictors to time to recovery from MAM among 6–59 months of children in Fedis Woreda, east Hararghe, Ethiopia, 2022.

Characteristics	Category	No	CHR (95%CI)	*p*-value	AHR (95%)
Person with primary Child responsibility	Mother	541	1	1	1
	Father	26	1.64 (0.92–3.3)	0.19	0.42 (0.1–1.04)
MUAC at Admission	11.5–12.0	340	1	1	1
	12.1–12.4	227	1.34(0.88–1.54)	0.21	1.20 (1.01–1.19)
Vitamin A	Yes	478	1	1	1
	No	89	0.81(0.64–1.02)	0.23	0.93(0.69–1.08)
Admission status	New	498	1	1	1
	Readmission	69	1.52(0.81–3.01)	0.18	1.28(0.6–1.72)
Transportation access to nearest health post and food distribution center	Yes	333	1	1	1
	No	234	0.73(0.52–0.89)	0.02	0.62(0.36–0.89)
Measles vaccination	Yes	396	1	1	1
	No	171	0.65(0.51–1.93)	0.15	1.21(0.73–1.46)
Specialized nutritious foods given to the child	CSB++	36	1	1	1
	RUSF	531	1.82(1.31–2.32)	0.01	1.96(1.36–3.11)
Fever	Yes	82	1	1	1
	No	485	2.1(0.92–7.45)	0.19	1.78(0.51–5.4)
Diarrhea	Yes	51	1	1	1
	No	516	1.33(0.93–2.01)	0.12	0.40(0.31–0.71)

The study conducted a multivariable Cox-regression analysis to identify predictors of the time to recovery for children with moderate acute malnutrition (MAM) enrolled in the Fedis Woreda targeted supplementary feeding program (Table 5). After controlling for confounding variables, the analysis yielded significant findings. Firstly, children admitted with a mid-upper arm circumference (MUAC) measurement of 12.1–12.4 centimeters had a 1.02 times higher likelihood of early recovery compared to those with MUAC between 11.5 and 12.0 centimeters. Secondly, families without access to transportation had a 38% lower likelihood of recovery compared to those with transportation access. Additionally, children who received ready-to-use supplementary feeding were 1.96 times more likely to recover within 16 weeks compared to those who received super cereal plus. Lastly, children with diarrhea had a 69% lower likelihood of recovery within 16 weeks compared to those without diarrhea. These findings emphasize the importance of factors such as MUAC at admission, transportation access, type of supplementary feeding, and the presence of diarrhea in influencing the time to recovery for children with MAM.

## Discussion

The study assessed the time to recovery from MAM and its predictors among children aged 6–59 months treated at a targeted supplementary feeding program at Fedis Woreda. The study found that the median time to recovery for children with moderate acute malnutrition (MAM) was 16 weeks. This duration aligns with the national standard malnutrition guidelines in Ethiopia, which also recommend a 16-week time frame (15). A similar study conducted in Darolebu reported a median recovery time of 16 weeks as well (27). However, in a study conducted in Shalla District, the median time to recovery was slightly lower at 15 weeks with an interquartile range (IQR) of 5 weeks (28). These comparisons provide a context for the recovery time observed in the current study, highlighting its consistency with national guidelines and other studies conducted in the region.

The study demonstrated an impressive overall recovery rate of 87.5%, surpassing the acceptable threshold set by the Sphere International Standard, which establishes a minimum recovery rate of 75% (29). This noteworthy achievement may be attributed to the regular follow-up provided by non-governmental organizations involved in the program as well as the continuous health education support delivered by health extension service workers at the local level. These factors likely played a crucial role in promoting successful recovery among the children with moderate acute malnutrition in the study.

The study findings revealed that children admitted with a mid-upper arm circumference (MUAC) measurement of 12.1–12.4 had a 1.02 times higher likelihood of early recovery within 16 weeks compared to children with MUAC between 11.5–11.9 (AHR = 1.02; 95% CI: 1.01–1.19). This observation aligns with previous studies conducted by James et al. and Kumsa and Silassie (18, 28). These studies collectively indicate that early screening for moderate acute malnutrition in children, coupled with the provision of supplementary feeding, leads to improved outcomes. Furthermore, the study found that children with the highest MUAC measurement at enrolment had a significantly reduced risk of remaining with moderate acute malnutrition and a higher chance of recovering. This highlights the importance of early detection and intervention in achieving better outcomes for children with moderate-acute malnutrition.

The study findings highlighted the significant impact of transport access to the nearest health facility providing targeted supplementary feeding services on the time to recovery. It was observed that families without transport access had a 38% lower proportion of children recovering from moderate acute malnutrition (MAM) compared to those with transportation access (AHR 0.62, 95% CI 0.36–0.89). This result is consistent with a study conducted in Ethiopia by Shanka et al. (30). The findings suggest that children who have access to transportation are more likely to receive timely and accessible services, leading to a better prognosis and improved recovery from MAM.

This study found that children who received ready-to-use supplementary food (RUSF) had a significantly higher likelihood of timely recovery from moderate acute malnutrition (MAM) within 16 weeks compared to those who received a corn-soy blend with oil (CSB++). The findings are consistent with studies conducted in Sidama Zone (31), Shala District (28), and Daro Labu District in Ethiopia, as well as in Malawi (32). These studies consistently demonstrate that children treated with RUSF had better recovery outcomes compared to those treated with CSB++. The sharing of CSB++ with other household members and the irregularity in daily cooking and cooking time were potential factors contributing to the lower recovery rates associated with CSB++. The absence of cooking demonstrations by governmental and non-governmental organizations at health posts may have contributed to a lack of awareness at the community level. Similarly, another study conducted in Jimma, Ethiopia, showed that MAM children treated with CSB+ and oil under the targeted supplementary feeding program (TSFP) had a lower rate of timely recovery compared to children treated with super cereals. Overall, the findings indicate that treating MAM children with ready-to-use supplementary food (RUSF) is more effective than using CSB++.

The study found that the presence of diarrhea in children with moderate acute malnutrition (MAM) admitted to the targeted supplementary feeding program (TSFP) had a significant impact on recovery outcomes. Children who had diarrhea were 69% less likely to recover within the 16-week timeframe compared to those who did not have diarrhea. This suggests that the presence of diarrhea acts as a barrier to timely recovery from MAM. By recognizing the association between diarrhea and delayed recovery in MAM children, healthcare providers can prioritize comprehensive care that targets both the nutritional needs and gastrointestinal health of these children.

This study is not without its own limitations. The study used the recorded data of the discharged children to determine the time-to-recovery from moderate acute malnutrition and identify its predictors among children aged 6–59 months who were admitted to the targeted supplementary feeding program in Fedis Woreda. As a result, this study was restricted to using medical records to determine time to recovery because it was not possible to include additional factors such as parental education and economic position, a history of breastfeeding, and other aspects. Another limitation of this study is the completeness of the health posts’ records. Additionally, factors like the wealth index, one of the determinants of malnutrition, are not evaluated.

## Conclusion and recommendations

In general, this study emphasizes the significance of early screening, access to transportation, appropriate supplementary foods, and effective management of diarrhea in determining the recovery outcomes of children with moderate acute malnutrition (MAM) within targeted supplementary feeding programs (TSFPs). Early detection and timely intervention, along with transportation access, play crucial roles in promoting timely recovery. The use of ready-to-use supplementary foods (RUSF) is associated with higher chances of recovery compared to corn-soy blends with oil (CSB++). However, the presence of diarrhea poses a significant barrier to recovery. By addressing these factors comprehensively, stakeholders can enhance recovery outcomes and reduce the burden of MAM in children.

The health extension workers should create public awareness and give health education on signs and symptoms of malnutrition that enhance early identification of the case and timely use of routine treatments. Early detection of co-morbidities like diarrhea is expected to reduce moderate acute malnutrition. Additionally, the woreda health office should work on expanding health facilities, building infrastructure like an all-season dry road, and supplying RUSF that will help the beneficiaries. Also, non-governmental organizations should provide technical and logistic support to health facilities working on TSFP for effective implementation and capacitate the health extension workers and health workers working on managing MAM, and they should ensure an adequate supply of RUSF and monitor its distribution to ensure proper utilization. Furthermore, the researcher should further examine how engagement with the community and caregivers through the implementation of nutrition education activities, counseling, and home visits contributes to successful program delivery and the improvement of intervention outcomes.

## Data availability statement

The raw data supporting the conclusions of this article will be made available by the authors, without undue reservation.

## Ethics statement

The studies involving humans were approved by Haramaya University’s Institutional Health Research Ethics Review Committee, with reference number C|AC|p1|D|a1|3,123|22. The studies were conducted in accordance with the local legislation and institutional requirements. Written informed consent for participation was not required from the participants or the participants’ legal guardians/next of kin in accordance with the national legislation and institutional requirements.

## Author contributions

BM: Formal analysis, Methodology, Writing – original draft, Writing – review & editing. BG: Conceptualization, Formal analysis, Investigation, Methodology, Writing – original draft, Writing – review & editing. BN: Conceptualization, Supervision, Writing – original draft, Writing – review & editing. MK: Conceptualization, Methodology, Supervision, Writing – original draft, Writing – review & editing. OK: Formal analysis, Investigation, Methodology, Writing – original draft, Writing – review & editing. JD: Formal analysis, Investigation, Methodology, Writing – original draft, Writing – review & editing. RK: Conceptualization, Formal analysis, Investigation, Methodology, Writing – original draft, Writing – review & editing. DF: Conceptualization, Formal analysis, Investigation, Methodology, Writing – original draft, Writing – review & editing.
